# Testing the social mindfulness paradigm: Longitudinal evidence of its unidimensionality, reliability, validity, and replicability in a sample of health care providers

**DOI:** 10.1371/journal.pone.0281738

**Published:** 2023-02-10

**Authors:** Tobias Altmann, Marcus Roth

**Affiliations:** Department of Psychology, University of Duisburg-Essen, Essen, Germany; Private University Schloss Seeburg: Privatuniversitat Schloss Seeburg, AUSTRIA

## Abstract

**Objectives:**

Social mindfulness is a relatively new concept in psychological research and is attracting increasing attention. Recent studies have provided evidence of its relevance with regard to prosocial behavior and empathy, but also concerning individual well-being and psychological health. In such studies, social mindfulness has been assessed using the social mindfulness paradigm by Van Doesum and colleagues, which is the standard measure of social mindfulness to date. However, evidence is scarce or lacking with regard to whether this measurement approach is unidimensional, whether it produces (test-retest) reliable and valid measurements, and whether its associations with personality and empathy are replicable.

**Methods:**

To test these assumptions, we assessed a sample of 265 participants currently working in health care professions on social mindfulness, several concepts of empathy, and the HEXACO personality dimensions longitudinally at two measurement occasions.

**Results:**

The results supported the assumption of unidimensionality of the measure. Partial support was found for its reliability, validity, and replicability. Test-retest reliability was acceptable, but the associations with personality and empathy turned out weaker than expected.

**Conclusions:**

The social mindfulness paradigm is an interesting approach toward understanding social mindfulness, meaning mindfulness of other people’s needs. Potential directions for the further development of the social mindfulness paradigm and its network of relations, especially to empathy, are discussed.

## Introduction

Social mindfulness is a comparably new concept in psychological research [1]. It has been described as a variation of general mindfulness that is focused not on one’s self, but instead on the social aspects of one’s personal interactions [1, 2]. Several approaches have been applied to measure general mindfulness, ranging from self-report questionnaires to behavioral measures such as breath counting [3]. By contrast, studies on social mindfulness use almost exclusively one assessment approach: the social mindfulness paradigm [1]. Despite the growing interest in the topic, basic assumptions about the paradigm, such as its unidimensional factor structure, (test-retest) reliability, validity, and the replicability of its associations with related constructs (such as empathy), have only partially been tested or have not been tested at all in previous studies.

The purpose of the present study was therefore to test these assumptions about the social mindfulness paradigm. Before presenting our study, we discuss (a) the theoretical conceptualization of social mindfulness, (b) its measurement, and (c) its associations with personality and the related construct of empathy as presented in previous studies.

### The concept of social mindfulness

As the root of social mindfulness, general mindfulness has been described as based on two components: first, the self-regulation of attention toward one’s current experiences, and second, curiosity about and acceptance of one’s current experiences [4]. As such, mindfulness means paying attention to and appreciating one’s mental states and awareness [5].

Social mindfulness, as the social adaptation of general mindfulness, has been defined as being mindful of others, meaning recognizing their present state (i.e., their needs, interests, wants) and acting accordingly [1, 2, 6]. More specifically, the current conceptualization of social mindfulness involves being mindful of the fact that other people have needs and wants and relates to the assumption that, in general, people prefer to make their own choices (if the situation allows for a choice). Hence, social mindfulness entails allowing others to choose for themselves (i.e., to fulfill their need for autonomy) instead of determining a setting for them [1]. For instance, at lunch in the cafeteria, say there are three apples left: one is green and two are red. Person A now gets to choose their apple, knowing that a Person B will choose theirs later on. We would call Person A mindful if they pick the red apple (of which there are two), leaving a choice (green or red apple) for the next person.

Accordingly, social mindfulness has been shown to be a relevant predictor of social behavior, such as reciprocal cooperative behavior by the second chooser [7] or even sharing one’s possessions [8]. It becomes even more relevant in terms of leadership [9]: for instance, when Person A is the mayor of a large city or the manager of a hospital and there are many Person Bs whose need satisfaction depends on Person A’s choices. The disposition to be mindful of the needs of others may then be a highly relevant trait.

### Measurement of social mindfulness

In their initial publication on the concept of social mindfulness, Van Doesum and colleagues [1] also presented a measurement paradigm basing on the choice paradigm used by Kim & Markus [10]. The paradigm by Van Doesum et al. [1] is the standard assessment approach to date and has been applied in a number of studies [e.g., 8, 9, 11]. This measurement paradigm can be described as an implicit behavioral test usually conducted on a PC. When taking the test, the participant is instructed to choose (i.e., select by clicking their mouse) one object for themselves in each trial. In each test trial, there are three similar objects: Two objects are identical and one is comparable but unique in some way. For instance, say there are three pens identical in design and differing only in color: Two are blue and one is green. The instructions continue by informing the participant that a second person will make their choice from the remaining objects after the participant has made their choice. If the participant chooses the unique item, the second person will no longer have a choice between two colors. Such a decision is interpreted as not socially mindful according to the paradigm [1, 2]. There are also several distractor trials—for instance, using four similar objects consisting of two pairs of identical objects (e.g., two blue pens and two green pens)—to hide the purpose of the assessment.

As previous research has shown, the standard choice among the three items in the absence of any further instructions or guidelines for behavior appears to be to choose the unique object [12]. The social mindfulness paradigm score (choosing objects knowing that a Person B will choose theirs from what is left) would therefore represent interindividual differences in the choice of the nonunique object, interpreted as the result of taking into account the other person’s assumed desire to choose for themselves and acting accordingly.

It may be noted that there are further developments of this same measurement approach [13] and a yet unpublished self-report questionnaire [14]. Nevertheless, the social mindfulness paradigm remains by far the most frequently applied measure of social mindfulness.

Returning to theoretical implications, the inevitable empirical limitations of having to rely on a single measurement approach are accompanied by theoretical difficulties, as already acknowledged by Van Lange & Van Doesum [6]. The uniqueness of the measure leads to an overly strong and even circular relation between concept and measurement and may result in equating the concept with its measurement. This difficulty has been discussed concerning other constructs in personality research, such as sensation seeking as defined by Zuckerman’s Sensation Seeking Scales [15], discussed in detail by Roth et al. [16]. In the present study, we deliberately focused on assessing the measurement paradigm of social mindfulness, and we remain cautious about making inferences about the construct itself.

### Associations with empathy and personality

To further describe the construct of social mindfulness, Van Doesum et al. [1] presented a series of studies on covariates. They suggested that social mindfulness would have links to personality traits and would have especially strong links to empathy, specifically emotion recognition, empathic concern, and perspective-taking. They argued that the propensity and ability to recognize another person’s states and needs, to take their perspective, and to act accordingly constitute the foundations of empathy and prosocial behavior. Both empathy and prosocial behavior are described as playing dominant roles in how a person represents the needs of others and in how these needs influence the person’s own (prosocial) behavior, hence, social mindfulness. In line with this reasoning, several conceptualizations of social mindfulness also refer to it as a form of low-cost prosocial behavior [6, 13, 17].

In light of this reasoning, previous studies have hypothesized that social mindfulness would be associated with the empathic concern subscale and the perspective-taking subscale of the Interpersonal Reactivity Index (IRI [18]) as well as with the Reading the Mind in the Eyes Test (RMET [19]). Van Doesum et al. [1] found moderate but significant associations with IRI empathic concern (*r* = .28) and IRI perspective-taking (*r* = .21), confirming the relatedness of the constructs. However, a second study by the same research group [2] found only weak to marginal relations with empathic concern (*r* = .08) and perspective-taking (*r* = .11), thus calling into question the conceptual links. In addition, they used the same measure (IRI) in both studies, so that it remains unknown whether their positive findings are related to empathy or only to this specific measure of empathy.

Also, Van Doesum et al. [1] found that social mindfulness was not significantly associated with the RMET (*r* = .03). The main issue in emotion detection tests such as the RMET may be the unrealistic testing material. The RMET presents participants with greyscale still photos of people’s eyes (and a small area around them). The ecological validity of such measures has long been questioned [20], and newer developments involve color and multimodal stimuli [21, 22]. In sum, social mindfulness’ relation to empathy is challenging to evaluate due to the different choices of empathy measures in previous studies.

Concerning its relation to general personality, social mindfulness has been studied in relation to the HEXACO personality dimensions as conceptualized by Ashton & Lee [23]. Van Doesum et al. [1] found that social mindfulness was primarily associated with the honesty/humility dimension (*r* = .32) as well as with the agreeableness dimension (*r* = .24) of the HEXACO model. The authors argued that the findings could be explained by conceptual overlap. Honesty/humility as well as agreeableness are socially sensitive concepts. They are related to respecting the other person and recognizing their needs, which is conceptually close to Van Doesum et al.’s phrasing of social mindfulness (as described above).

However, the same research group [2] also found considerably lower associations with the aforementioned dimensions. When using the same HEXACO model, honesty/humility was then found to be only weakly related (*r* = .23 and .12 in Study 3 and 4), and agreeableness was weakly (*r* = .13 in Study 3) and not related (in Study 4) to social mindfulness. Using the Big Five model [24], only agreeableness was (weakly) related to social mindfulness (*r* = .08 in Study 1). It must be noted that in Study 4, there were six years between the assessments of the HEXACO and social mindfulness, potentially explaining the lower scores. However, focusing only on honesty/humility, Mischkowski et al. [25] also found the correlation with social mindfulness to be weak, with *r* around .10 and varying slightly depending on how the SoMi score was calculated.

Summarizing these findings, it seems questionable not only how strong, but first and foremost, how reliable social mindfulness’ associations with empathy and personality really are. Three issues may be relevant here. First, looking into the details of the studies, the diversity of previous findings as summarized above may be caused by differences in the quality of the samples. The assessments vary between interviewing participants at home (i.e., in the complete absence of anonymity) and using anonymous MTurk participants. Second, the results may also vary within samples over time. However, no study assessed the same sample twice to assess the stability of these associations or to check whether the scores produced by the instrument were at all stable over time. Third, the instructions (as detailed above) included an ostensible second person who would be choosing an item from what was left after the participant chose one item for themself. This alone might trigger not only social mindfulness [26], but also socially desirable response patterns (see especially Study 3 by Paulhus [27]). This issue may well be relevant in light of the different assessment types (interviewed individually vs. MTurk participants).

### The present study

The goal of the present study was to examine the social mindfulness measure for its unidimensionality, reliability, validity, and the replicability of previous and the present findings. To test for unidimensionality and reliability, we used confirmatory factor analysis, internal consistency, and split-half reliability at two measurement occasions, as well as test-retest reliability. Concerning validity, previous research has been inconclusive about the links to empathy and personality. To address previous shortcomings, we used a wider and updated selection of measures of empathy as well as two measurement occasions with the same sample to more rigorously assess the replicability (or temporal stability) of the findings.

We expected to find sufficient evidence of unidimensionality and reliability. We expected to find positive correlations with empathy, agreeableness, and honesty/humility. We expected to find stability or replicability of these associations using the same sample.

We used one-tailed significance tests to test our directional hypotheses. We also tested the relation between social mindfulness and socially desirable responding exploratorily to exclude alternative explanations.

## Method

### Procedure

Participants were recruited from among healthcare professionals at the university hospitals in Bonn, Cologne, Duesseldorf, and Essen, Germany. Data were collected in supervised group sessions of eight to 15 people. Questionnaires were administered in a paper-and-pencil format, and tests were conducted on PCs. Participation was voluntary and took place during participants’ working hours. Participants provided written informed consent prior to their participation.

All procedures were in accordance with the local ethical guidelines of all institutions involved. Three ethics committees approved all aspects of the study: The “Ethikkommission der Abteilung Informatik und Angewandte Kognitionswissenschaft der Fakultät für Ingenieurwissenschaften der Universität Duisburg-Essen” (no reference number given by the committee), the “Ethikkommission der Medizinischen Fakultät der Universität zu Köln” (no reference number given by the committee), and the “Ethik-Kommission–Medizinische Fakultät Bonn” (reference number: 154/16). The data used in the present study are available online at https://osf.io/a3cby/. The present study is part of a larger research project, of which the hypotheses and data presented here are one component.

All measures except for the HEXACO and the KSE-G (for details, see below) were assessed again 3.9 months after the first assessment. The second assessment used the same sample (except for individual dropouts) and paralleled the first assessment in all respects. Both the HEXACO and KSE-G have been shown to be and are conceptualized as temporally stable [28, 29] and were therefore not assessed a second time.

### Sample

The sample comprised 265 participants (81.8% women). The mean age was 38.6 years (*SD* = 11.4), ranging from 20 to 62 years. A total of 64.9% had higher secondary education (12 years at school) and the remainder lower secondary education (8–10 years at school). The mean number of years spent working in the participants’ present occupation was 17.0 years (*SD* = 11.2). A subsample of 232 participants completed the emotion recognition test (for details, see below). We decided to use only complete data sets (instead of imputing data); therefore, sample sizes vary between analyses and will be provided for each calculation.

Contrary to previous findings [30, 31], health care providers did not differ in their general prosocial scores compared to more representative samples from the general population. The scores of social mindfulness (SoMi, see details below) in the two measurement occasions in the present sample were 0.61 and 0.63 (*SD* = 0.19 and .22) and did not differ from to the SoMi score of .60 (*SD* = .22) described by Van Doesum et al. [1] using samples from the general population (comparisons with the two measurement occasions of the present sample: *t*(265 / 215) = 0.95 / 1.75, *p* = .345 / .082). Similarly, the sum scores of the TEQ (measure of empathy, see details below) were 44.2 and 45.4 (*SD* = 5.4 to 5.9) in the present sample and thus fall within the range of scores described by Spreng et al. [32] of 43.5 between 48.9 (*SD* ranging between 6.8 to 8.2) using samples from the general population.

As mentioned above, empathy scores have been found to differ between health care providers and the general population in previous studies [30, 31]; however, such studies predominantly assessed trainees (e.g., nursing students). Criticizing this, Paola et al. [33] found the empathy scores of nursing students to be significantly higher than those of fully trained nurses. This might explain why our sample of health care providers (trained professionals working in the field) did not differ from the general population. In conclusion, we do not expect any particular limitations in variance or range due to the sample that may have limited the potential associations.

### Measures

#### Social mindfulness

We used the same paradigm and procedure to assess social mindfulness as described by Van Lange & Van Doesum [6], using the implicit behavioral PC test format. The present test consisted of 24 trials: 12 test trials and 12 distractor trials. As described above, in each test trial, participants saw a set of objects (e.g., pencils) on a PC screen. All of the presented objects were identical except for one, which was comparable but slightly different, for instance, in color (for sample trials, see Van Lange & Van Doesum [6]). Participants were instructed to choose one object for themselves by clicking on it using the PC mouse. Participants were informed that afterward, a second person would be choosing from the objects that were left. Participants were not able to see or interact with the second person (who did not actually exist). In the distractor trials, four objects in two identical sets (e.g., two blue and two green pencils) or three completely identical objects were presented. As described above, if a participant selects one of the several identical items in a test trial, this choice is counted as a socially mindful choice (scored as 1); if the participant selects the unique item, this is counted as a not socially mindful choice (scored as 0). The SoMi score is calculated as the mean across all test trials. Details on the psychometric properties are provided in the Results section.

Apart from this standard calculation of the SoMi score, we also calculated the “consequence-adjusted SoMi score” following the procedure presented by Mischkowski et al. [25], which controls for object preference. As the authors describe it, the advantage of this score is that it “provides an indicator of how behavior changes in the presence of social consequences which mirrors the essence of social mindfulness” (p. 92). If a person chooses a red apple in the distractor trial and also chooses a red apple as the non-unique object in the test trial (i.e., a socially mindful option), both choices may potentially be motivated by a preference for this particular object or colour, etc., instead of–in the test trial–social mindfulness. To reduce this bias, this test trial is omitted from the calculation of the SoMi score for this person. This principle is applied to all objects with parallel objects in test and distractor trials. The standard SoMi score and the adjusted SoMi score correlated with *r* = .76, *p* < .001 and *r* = .72, *p* < .001 at the two measurement occasions.

#### HEXACO

The HEXACO dimensions were assessed with the German translation of the 60-item version of the HEXACO-PI-R [34, 35]. The HEXACO-PI-R contains six dimensions (Cronbach’s alpha in the present study in parentheses): Honesty/Humility (.68), Emotionality (.72), Extraversion (.69), Agreeableness (.67), Conscientiousness (.66), and Openness to Experience (.70). All items were rated on a 5-point scale ranging from 1 (totally disagree) to 5 (totally agree).

### Emotional Contagion Scale (ECS)

The Emotional Contagion Scale (ECS; [36]) is a self-report questionnaire for assessing the propensity to pick up on others’ emotions in five domains (love, happiness, sadness, anger, and fear). People high on emotional contagion are “those who pay close attention to others and are able to read others’ emotional expressions” (p. 133). Participants rated their agreement with the 15 items on a 5-point rating scale. In the present study, Cronbach’s alpha was .77.

### Geneva Emotion Recognition Test (GERT)

The Geneva Emotion Recognition Test (GERT; [22]) is a test for assessing the ability to correctly detect emotions in humans. In each trial, a short video of around three seconds is presented in which a person (one of several professional actors of both sexes and different ages) in front of a black background says a few made-up words. The words are the same in all video clips, but while saying the words, the person expresses one of 14 specific emotions. The participants are instructed to watch each video and to indicate afterward which emotion was portrayed. In the present study, we used the short version with 42 trials [37]. Cronbach’s alpha was .92.

*Toronto Empathy Questionnaire (TEQ)*. The Toronto Empathy Questionnaire (TEQ [32]) is a 16-item self-report measure of empathy. The TEQ has been used in different contexts and countries (for an overview, see Kourmousi et al. [38]). In the present study, agreement with the items was rated on the same 5-point rating scale as above. Cronbach’s alpha was .59 (95% CI [.51, .66]).

### Self-Reported Perspective-taking (PT)

We adapted three items from the Trait Emotional Intelligence Questionnaire [39] to assess the perspective-taking facet of empathy. The instructions asked participants to rate their propensity “to put yourself in somebody else’s shoes,” “to conceive of somebody’s emotions,” and “to conceive of somebody’s needs.” Agreement with the items was rated on the same 5-point rating scale as above. Cronbach’s alpha for the three items was .68.

### Socially Desirable Responding (KSE-G)

We used the Short Scale of Social Desirability-Gamma (KSE-G [29]). Using the same 5-point rating scale to measure agreement as above, the KSE-G assesses the following two dimensions with three items each: enhancement of positive qualities (Cronbach’s alpha = .45; 95% CI [.32, .56]) and denial of negative qualities (Cronbach’s alpha = .42; 95% CI [.28, .53]). The internal consistency was below the acceptable level; therefore, the results should be interpreted with caution [29].

## Results

### Factor structure and reliability

We first tested the factor structure for the assumed unidimensionality at each measurement occasion. Confirmatory factor analyses using the overall fit measures Root Mean Square Error of Approximation (RMSEA) and Standardized Root Mean Square Residual (SRMR) were conducted using the R package lavaan [40]. We found sufficient fit for the single latent factor model at both the first and the second measurement occasions (χ^2^ = 107.5 / 77.4, *p* < .001 / = .020, RMSEA = .062 / .046, SRMR = .066 / .060). As futher evidence, we calculated internal consistency scores of the social mindfulness paradigm using Cronbach’s alpha with 95% confidence intervals. At the first measurement occasion and based on 265 participants, Cronbach’s alpha was .53 (95% CI [.45, .61]), with corrected item-total correlations ranging from .06 to .34 (*M* = .21). At the second measurement occasion and based on 216 participants, Cronbach’s alpha was .68 (95% CI [.61, .74]), with corrected item-total correlations ranging from .14 to .45 (*M* = .32). A detailed look into potential changes in Cronbach’s alpha at the item level did not reveal any specific items that severely reduced the score or would have enhanced it if this item had been excluded. However, when rank ordering the items based on their corrected item-total correlation, one item scored consistently low and also occupied the last rank at both measurement occasions (item number 4 in the item list provided by Van Lange and Van Doesum on their social mindfulness webpage, https://www.socialmindfulness.nl/s/SoMi_pictures.zip).

Concerning reliability, we assessed split-half reliability at both measurement occasions and test-retest reliability across the two measurement occasions. Split-half reliability was calculated using the odd-even method (i.e., allocating every second item to the first half of the test and every other item to the second half of the test and correlating the two). For the first measurement occasion and based on 265 participants, the split-half reliability was .47 (95% CI [.37, .56]), (*p* < .001). For the second measurement occasion and based on 216 participants, the split-half reliability was .51 (95% CI [.41, .61]), (*p* < .001).

The calculation of test-retest reliability was based on the subsample of 162 participants who took part in both measurement occasions. Its Pearson correlation coefficient was .52 (95% CI [.40, .63]), (*p* < .001), and its intraclass correlation coefficient ICC(2,1) [41] was .67 (95% CI [.57, .75]), (*p* < .001). Both test-rest reliability scores are consistent with the internal consistency and split-half reliability scores. Using the adjusted score, test-retest reliability was .33 (95% CI [.21, .45]) and ICC(2,1) was .50 (95% CI [.34, .61]).

To account for the low internal consistency and split-half reliability scores, we also calculated the adjusted or disattenuated test-retest correlations using the Spearman formula as described by Zimmerman & Williams [42]. We used the internal consistency scores as well as split-half reliability for both measurement occasions and calculated the disattenuated test-retest reliability for each. The resulting disattenuated scores ranged from .87 to .99.

### Validity and replicability

We then tested the associations between social mindfulness and the HEXACO dimensions and facets at both measurement occasions. In Table 1, we juxtapose our results with the previous findings presented by Van Doesum et al. [1], who analyzed a sample of 186 participants. On the dimension level, only the association with the HEXACO dimension of honesty/humility appeared replicable (i.e., there was a significant correlation in the previous study and at both measurement occasions in our study). On the facet level, the same was true only for modesty, as a facet of the honesty/humility dimension. All correlation coefficients remained almost identical when controlling for age and sex.

**Table 1:** Correlations between social mindfulness scores (First and second measurement occasions) and HEXACO dimensions and facets (First measurement occasion).

	Social mindfulness		*Van Doesum et al*. [1]
	t1	t2	
**Honesty/Humility**	.20***	.12*	.32^***^
Sincerity	.16**	.17**	.*12*
Fairness	.10*	.02	.19^**^
Greed avoidance	.06	.02	.25^**^
Modesty	.21***	.12*	.30^***^
**Emotionality**	-.01	.07	.*13*
Fearfulness	-.14*	-.05	*-*.*01*
Anxiety	-.04	-.01	.*09*
Dependence	.10*	.12*	.*07*
Sentimentality	.07	.12*	.23^**^
**Extraversion**	.08	.02	*-*.*05*
Social self-esteem	.13*	.08	*-*.*06*
Social boldness	.02	.01	*-*.19^*^
Sociability	.03	-.02	.*08*
Liveliness	.00	-.05	.*04*
**Agreeableness**	.04	.05	.24^***^
Forgiveness	.07	.01	.15^*^
Gentleness	.05	.04	.20^**^
Flexibility	-.01	.05	.21^**^
Patience	.01	.04	.16^*^
**Conscientiousness**	.01	.12*	.*05*
Organization	-.04	-.01	*-*.*05*
Diligence	-.04	-.05	.*12*
Perfectionism	-.01	.08	.*07*
Prudence	.08	.23***	.*02*
**Openness to Experience**	.00	.01	.*05*
Aesthetic appreciation	-.09	.01	.*10*
Inquisitiveness	.03	.02	*-*.*09*
Creativity	.00	-.01	.*05*
Unconventionality	.06	.01	.*08*

*Note*. t1 = first measurement occasion, t2 = second measurement occasion. Sample sizes were *N* = 265 at t1 and *N* = 216 at t2.

* *p* < .05.

** *p* < .01.

*** *p* < .001.

The correlation coefficients remained largely the same when using the adjusted SoMi score rather than the classic SoMi score (see Method section). The largest differences between the classic vs. adjusted SoMi scores (albeit still representing small effects in both cases) were found with regard to honesty/humility and its facets for the first measurement occasion: honesty/humility *r* = .20, *p* < .001 / *r* = .25, *p* < .001, sincerity *r* = .16, *p* = .005 / *r* = .15, *p* = .007, fairness *r* = .10, *p* = .046 / *r* = .17, *p* = .003, greed avoidance *r* = .06, *p* = .150 / *r* = .17, *p* = .003, and modesty *r* = .21, *p* < .001 / *r* = .17, *p* = .002.

Table 2 presents the results of the correlations between social mindfulness and aspects of empathy. Neither the TEQ as a general measure of (affective) empathy (which includes several items from the IRI empathic concern subscale) nor the PT measure of perspective-taking were significantly correlated with social mindfulness. These findings are in contrast to the previous findings by Van Doesum et al. [1]. Also in contrast to the previous study, the GERT measure of emotion recognition was significantly correlated with social mindfulness at both measurement occasions. Extending the scope of empathy by including the ECS as a measure of emotional contagion did not yield a better understanding of social mindfulness because this correlation was nonsignificant. Across all correlations, the coefficients in our study remained almost identical when controlling for age and sex.

**Table 2:** Correlations between social mindfulness scores (First and second measurement occasions) and measures of empathy (First and second measurement occasions).

		Social mindfulness		*Van Doesum et al*. [1]
		t1	t2	
ECS	t1	.06	-	*-*
	t2	-	.09	
GERT	t1	.18**	-	*RMET*: .*03*
	t2	-	.21***	
TEQ	t1	.10^x^	-	*IRI-EC*: .28^***^
	t2	-	.04	
PT	t1	-.00	-	*IRI-PT*: .21^**^
	t2	-	.00	

*Note*. t1 = first measurement occasion, t2 = second measurement occasion. Sample sizes ranged from 193 to 265. RMET = Reading the Mind in the Eyes Test revised version [19]; IRI = Interpersonal Reactivity Index [18]; IRI-EC = IRI subscale Empathic Concern; IRI-PT = IRI subscale Perspective-taking.

^x^ *p* < .10.

* *p* < .05.

** *p* < .01.

*** *p* < .001.

The correlation coefficients remained largely the same when using the adjusted SoMi score rather than the classic SoMi score (see Method section). The largest changes (albeit still representing small effect sizes in both cases) were found at the second measurement occasion with regard to the GERT (correlation with the classic SoMi score *r* = .21, *p* < .001, correlation with the adjusted SoMi score *r* = .15, *p* = .015) and with regard to the TEQ (correlation with the classic SoMi score *r* = .041, *p* = .271; correlation with the adjusted SoMi score *r* = .13, *p* = .029).

The third aspect of validity we tested in this study was the relation between social mindfulness and socially desirable responding. Social mindfulness did not yield any significant correlations with either enhancement of positive qualities (for t1/t2: *r* = .00/.02) or denial of negative qualities (for t1/t2: *r* = .02/.05). Hence, the test of (partial) mediation of the relation between social mindfulness and empathy was not conducted.

## Discussion

The goal of the present study was to examine the social mindfulness paradigm for the psychometric characteristics indicative of a personality trait. Previous research in this regard, i.e., testing the paradigm with regard to its replicable validity, unidimensionality, and reliability, has been scare if not lacking.

### Discussion of validity and replicability

With respect to validity and replicability, we found our results to only partial support the assumption of replicability of findings of previous studies. The most stable finding was the positive relationship with the HEXACO dimension of honesty/humility, and therein the facet of modesty. Results were slightly higher for honesty/humility and most of its facets when using the preference-adjusted score [25], but only in the first of the two measurement occasions. Interpreting these replicable findings, we find that social mindfulness may be seen as related to treating others respectfully, containing one’s impulses, being modest and unassuming, and viewing oneself as an ordinary person without any claim to special treatment.

The association between the social mindfulness paradigm and agreeableness could not be replicated with either of the two measurements of agreeableness used in our study. Failure to find this expected association was also reported by Van Doesum et al. [2], questioning this link. Creating a stable nomological network around social mindfulness thus seems to be a still-unresolved challenge.

Furthermore, despite its obvious positive connotation, social mindfulness did not seem to be influenced by socially desirable responding, making it easily applicable in research and practice. This finding also supports the assumption that participants are unaware of what is actually being measured by the paradigm.

We also found a positive association with a test of ability to detect emotions in other people, linking social mindfulness to at least some aspects of empathy. However, when self-report questionnaires (instead of tests) were used to assess empathy, we did not find a significant correlation with social mindfulness. A small correlation was found, but only with the TEQ and only when using the preference-adjusted SoMi score [25]. There are several rationales which might contribute to explaining these findings.

First, this lack of association may in part be due to general problems with self-report questionnaires for empathy. Self-report measures of empathy tend to be only weakly correlated with one another [43] and only marginally correlated, if at all, with behavioral measures of empathy [44]. They also tend to show low levels of self-other agreement [45], thus calling their validity into question. In contrast, behavioral measures assess actual behavior, so their ecological validity can be assumed to be superior to (or complement) self-reports. The typically used forced-choice formats of behavioral measures (also used in the SoMi) better capture the challenges of everyday life in which people often have to make dichotomous decisions (as discussed by Van Lange et al. [46]), such as decide whether to stop their car at or drive by a road accident.

Second, an alternative explanation for the lack of association between the SoMi and the applied self-report measures may be methodological. The SoMi is a nonverbal behavioral measure of choices about pictures. Thus, it does not rely on language, in stark contrast to verbal self-report questionnaire measures. This difference alone may contribute to explaining the lack of association between SoMi and the applied self-report measures [46].

Third, in light of the complexity of the concept of empathy [47, 48], which has been debated for decades [49], social mindfulness might be more relevant to (or even consist of) prosocial behavior [6], rather than empathy. Empathy surely can be seen as a catalyst of prosocial behavior as assessed by the social mindfulness paradigm, but it is hardly a necessary condition for it. Attempts to distinguish the empathy process [50] from prosocial motivation and potential outcomes of empathy [51, 52] might help researchers focus on more potential covariates of social mindfulness. In line with this reasoning, Van Doesum et al. [53] studied prosociality among people from different social classes using the SoMi and a “dictator game” as a measure of prosociality (the “dictator” or decision-maker decides how much of a limited resource, such as money or points, etc., they and the other person will receive). They found replicable results showing higher prosociality scores among people from lower social classes.

Lastly, it might be interesting in future research to study the relationship between social mindfulness and the quality of responses in empathic interactions (i.e., interactions in which one person shares personal emotional information with another person). It can be hypothesized that people higher in social mindfulness are less likely to use empathic short-circuit responses [47] and are more likely to use responses that validate the experiences and feelings of the other person. In any case, the relations between social mindfulness and empathy remain speculative given the present state of knowledge.

### Discussion of reliability

We also found mixed results concerning reliability. For internal consistency, the confidence intervals for Cronbach’s alpha for the two measurement occasions were adjoined but did not overlap, with the alpha score from the second measurement occasion being substantially higher. This might indicate that internal consistency was higher for participants with prior experience with the paradigm, calling into question the stability of the assessment to some extent. In general and compared with the Cronbach’s alpha scores that are common in personality assessment, the score in the first assessment was quite low considering the often used threshold of .70 [54]. However, some scholars have also suggested accepting lower scores as sufficient for group comparisons and correlational analyses across samples [55, 56].

More generally, using internal consistency as a criterion for scale quality has been a matter of much debate [57]. The uses and misuses of internal consistency measures as an indicator of reliability have been widely discussed but also widely ignored [58, 59]. Cronbach’s alpha may be less relevant as a criterion of scale quality [57] in cases where the score represents a broad concept.

This is also true for the SoMi, as it includes a variety of objects (e.g., cookies, baseball caps, flowers, etc.) in various shapes and colors which may or may not have different personal meanings for different participants. Thus, the internal consistency of a scale may necessarily be lower (without compromising the scale’s overall quality) when a large range of different items have to be included to represent such a complex construct in its entirety.

The split-half reliability scores from the two measurement occasions in our study were comparably high. However, similar to the internal consistency scores, they were below the level common among most personality measures. In light of these scores, the split-half reliability of the measurement paradigm can be deemed acceptable, at least for measuring group differences in a research context [60].

After controlling for internal consistency and split-half reliability, the test-retest reliability score was very high, comparable to or even above those commonly found for intelligence tests (deemed the most reliable personality tests). Again, these findings show that low internal consistency scores (and relatedly, split-half reliability) do not necessarily indicate an insufficient measurement approach.

### Limitations

Two major limitations of the present study have to be mentioned. First and foremost and also as discussed in the Introduction, the social mindfulness paradigm is the sole standard approach for assessing the construct to date (recent advancements include Engel and Van Lange [13]). Therefore, the conceptualization and measurement of the construct were confounded in the present evaluation. We deliberately focused our evaluation on the measurement so that our conclusions are data-driven. Thus, the present findings refer to the assessment of social mindfulness and not the concept as such. Consequently, additional interpretation of the present findings, that is, that social mindfulness as a concept qualifies as a stable trait, needs to be made with caution. Other methods for assessing social mindfulness need to be developed to locate and validate the concept in a multitrait-multimethod matrix [61].

In addition to further developments of the SoMi measure (e.g., Engel & Van Lange [13]), a current but yet unpublished approach [14] for assessing the construct using a self-report questionnaire seems promising and may be a potential candidate with respect to construct validity. However, other constructs in personality research have also been translated into different assessment formats (i.e., self-reports, tests, or objective or behavioral measures), commonly leading to inconclusive interrelations (e.g., empathy and intelligence; [44, 62]). The same is true for general mindfulness, which, as described above, has been proposed to be the starting point for the development of social mindfulness. In general mindfulness as well, self-reports and objective measures tend to be weakly correlated at best [63]. In addition to this tendency, self-report questionnaires are typically influenced by socially desirable responding, which does not appear to be the case for the social mindfulness paradigm, as evidenced in the present study.

A second limitation refers to the composition of the sample. We used a sample of healthcare professionals, who are typically predominantly female. Although the present sample offers a satisfactory age range and different educational backgrounds, it is predominantly female, which may account for the different results found in the present study compared to those previously published. In our analyses, we controlled for sex (in addition to age) and found that the results remained practically unaltered. We also found that the present sample did not differ from more general samples with regard to their social mindfulness scores and empathy scores (in contrast to previous findings on nursing students exhibiting higher empathy scores than the general population [30, 31], as described in the Method section). However, the sample may differ from more general samples in other respects, such as self-addressed expectations about their own empathy and pro-sociality, as well as other factors such as burnout. Thus, the specificity of the present sample may have affected the results and may limit their generalizability.

### Theoretical and practical implications

In sum, the social mindfulness paradigm shows promising psychometric qualities. Its independence of socially desirable response tendencies and its satisfactory test-retest reliability make it a measure of high potential, despite concerns about its internal consistency (discussed above). Of course, different samples than the present sample of healthcare providers are needed to validate and extend the present findings.

With regard to validity, we found partial replicability. However, empathy and the HEXACO model of personality might not be the best choices to evaluate the social mindfulness paradigm’s validity in the first place. Instead, future research might seek to study the relations between social mindfulness and outcomes of empathy, such as the quality of individual responses and conversational partners’ satisfaction with these responses in contexts such as leadership [9] or psychotherapy.
